# Supplementary material for: Data mining and spatio-temporal characteristics of urban road traffic emissions: A case study in Shijiazhuang, China
Source: PLoS One. 2023 Dec 13;18(12):e0295664. doi: 10.1371/journal.pone.0295664 (PMC10718443; doi:10.1371/journal.pone.0295664)
Supplement: S1 Table — (DOCX) [file pone.0295664.s002.docx]

**S1 Table.** Attribute information of nodes and edges in topology

| Attribute category | Attribute filed | Data types | Description |
| --- | --- | --- | --- |
| Node | ID_P | int | The node ID is the unique identification of a intersection |
|  | Name_P | string | Intersection name |
|  | Longitude | double | Longitude of Intersection |
|  | Latitude | double | Latitude of intersection |
| Link | ID_E | int | The link ID is the unique identification of the road |
|  | Name_E | string | Road Name |
|  | F_PointID | int | ID of the start of the road |
|  | T_PointID | int | ID of the end of the road |
|  | Length | double | Road geometry |
|  | Two-way | int | 0 means the road is one-way, 1 means the road is two-way |
|  | Lane number | int | Describes the number of lanes |
