# Supplementary material for: Data mining and spatio-temporal characteristics of urban road traffic emissions: A case study in Shijiazhuang, China
Source: PLoS One. 2023 Dec 13;18(12):e0295664. doi: 10.1371/journal.pone.0295664 (PMC10718443; doi:10.1371/journal.pone.0295664)
Supplement: S2 Table — (DOCX) [file pone.0295664.s003.docx]

**S2 Table.** Line event information in linear reference models

| **Event type** | **Attribute filed** | **Data types** | **Description** |
| --- | --- | --- | --- |
| Basic properties of line events | Feature | string | Linear Reference Elements |
|  | LID | string | The ID is the unique identification of the edge |
|  | F_PointID | double | ID of the start of the road |
|  | T_PointID | double | ID of the end of the road |
| Line Event Traffic Information | Time_P | string | Time label, indicating the moment of traffic data |
|  | A_Speed | double | Average speed (km/h) |
|  | A_Time | double | Travel time (s) |
|  | A_Distance | double | Travel distance (m) |
