# Supplementary material for: Data mining and spatio-temporal characteristics of urban road traffic emissions: A case study in Shijiazhuang, China
Source: PLoS One. 2023 Dec 13;18(12):e0295664. doi: 10.1371/journal.pone.0295664 (PMC10718443; doi:10.1371/journal.pone.0295664)
Supplement: S3 Table — (DOCX) [file pone.0295664.s004.docx]

**S3 Table. Roads with high traffic emission intensity**

| **Road Name** | **Id** | **Road Type** | **Start Location** | **End Location** |
| --- | --- | --- | --- | --- |
| South 2^nd^ Ring West Road | 1 | Expressway | Intersection of West 2^nd^ Ring Road and South 2^nd^ Ring Road | Intersection of Shitong road and South 2^nd^ Ring Road |
| Shitong Road | 2 | Secondary | Intersection of West 3^rd^ Ring Road and Shitong Road | Intersection of Shitong road and South 2^nd^ Ring Road |
| Yuquan Road | 3 | Expressway | Intersection of West 3^rd^ Ring Road and Yuquan Road | Intersection of West 2^nd^ Ring Road and South 2^nd^ Ring Road |
| Xinhua Road | 4 | Secondary | Intersection of West 3^rd^ Ring Road and Xinhua Road | Intersection of West 2^nd^ Ring Road and Xinhua Road |
| West 3^rd^ Ring Road | 5 | Expressway | Intersection of West 3^rd^ Ring Road and Xinhua Road | Intersection of West 3^rd^ Ring Road and Zhongshan Road |
|  | 6 | Expressway | Intersection of West 3^rd^ Ring Road and Yuhua Road | Intersection of West 3^rd^ Ring Road and Huai'an Road |
|  | 7 | Expressway | Intersection of West 3^rd^ Ring Road and Huai'an Road | Intersection of West 3^rd^ Ring Road and South 2^nd^ Ring Road |
| Huitong Road | 8 | Secondary | Intersection of Jiefang South Street and South 2^nd^ Ring Road | Intersection of Pingan South Street and Huitong Road |
|  | 9 | Secondary | Intersection of Pingan South street and Huitong Road | Intersection of Jianshe South Street and Huitong Road |
| Jiefang South Street | 10 | Trunk | Intersection of Jiefang South Street and South 2^nd^ Ring Road | Intersection of Jiefang South Street and Huaian Road |
