# Supplementary material for: Data mining and spatio-temporal characteristics of urban road traffic emissions: A case study in Shijiazhuang, China
Source: PLoS One. 2023 Dec 13;18(12):e0295664. doi: 10.1371/journal.pone.0295664 (PMC10718443; doi:10.1371/journal.pone.0295664)
Supplement: Attachment — Submitted filename: Comment set.docx [file pone.0295664.s005.docx]

Comments Set 1:

This paper introduces a methodology for using big data sources to estimate emission intensity on roadways. The proposed methodology can replace the expensive methods of evaluating roadway emissions. Based on my review of this paper, here are my comments:

(1) Line 181 In equation 3, why is emission intensity directly related to length? What does this relationship mean? What is the unit of "Hp g/veh-km"? If the average speed is the same, would a road with a 2 km link have double the emission intensity of a road with a 1 km link?

(2) Line 87: Since traditional GPS-based emission analysis requires heavy data processing and specialized training, how easy is it to use the proposed method? If decision-makers intend to use the proposed methodology, how many days would it take to complete the analysis?

(3) Line 38: In the abstract, mention the actual number or percentage of segments on Second and Third Street instead of "some segments."

(4) Line 171: Please cite the original data source for Table 1.

(5) Line 208: What percentage of the traffic data was original, and what percentage of data was missing that required linear interpolation?

(6) Line 211: Provide further details on how Equation 4 was used in the case study. Was the average speed for every 15 minutes used?

(7) Figures 6-9: Why is CO emission almost constant regardless of the time of day? Is CO emission not affected by slowdowns during peak hours?
